# The Golgi complex: An organelle that determines urothelial cell biology in health and disease

**DOI:** 10.1007/s00418-022-02121-0

**Published:** 2022-06-30

**Authors:** Mateja Erdani Kreft, Alexander A. Mironov, Samo Hudoklin

**Affiliations:** 1 Faculty of Medicine, Institute of Cell Biology, University of Ljubljana, Ljubljana, Slovenia; 2 The FIRC Institute of Molecular Oncology, Milan, Italy

**Keywords:** Golgi complex, Blood–urine barrier, Urothelium, Differentiation, Bladder cancer, Uroplakins

## Abstract

The Golgi complex undergoes considerable structural remodeling during differentiation of urothelial cells in vivo and in vitro. It is known that in a healthy bladder the differentiation from the basal to the superficial cell layer leads to the formation of the tightest barrier in our body, i.e., the blood–urine barrier. In this process, urothelial cells start expressing tight junctional proteins, apical membrane lipids, surface glycans, and integral membrane proteins, the uroplakins (UPs). The latter are the most abundant membrane proteins in the apical plasma membrane of differentiated superficial urothelial cells (UCs) and, in addition to well-developed tight junctions, contribute to the permeability barrier by their structural organization and by hindering endocytosis from the apical plasma membrane. By studying the transport of UPs, we were able to demonstrate their differentiation-dependent effect on the Golgi architecture. Although fragmentation of the Golgi complex is known to be associated with mitosis and apoptosis, we found that the process of Golgi fragmentation is required for delivery of certain specific urothelial differentiation cargoes to the plasma membrane as well as for cell–cell communication. In this review, we will discuss the currently known contribution of the Golgi complex to the formation of the blood–urine barrier in normal UCs and how it may be involved in the loss of the blood–urine barrier in cancer. Some open questions related to the Golgi complex in the urothelium will be highlighted.

## Introduction

The formation of an effective blood–urine barrier, which is the tightest epithelial barrier in the body, is of fundamental importance for bladder function and metabolic homeostasis of the terrestrial mammalian species (Hicks 1975). This barrier would not be possible without the specific molecular and morphological adaptations of the urothelium, which lines the renal pelvis, ureters, urinary bladder, and proximal urethra (Romih et al. 2005). The urothelium has two possible permeation pathways (Lasič et al. 2015). The transcellular pathway consists of the apical and basolateral plasma membranes. The main transcellular permeability barrier is the apical plasma membrane of superficial urothelial cells (UCs), which is characterized by numerous specialized features, such as its superficial glycosaminoglycan layer (Parsons 2007), its particular lipid composition (Grasso and Calderón 2009; Resnik et al. 2015) and foremost urothelial plaques composed of the transmembrane proteins uroplakins (UPs) (Yu et al. 1990; Wu et al. 1990; Hu et al. 2002, 2000; Kachar et al. 1999), all of which influence the course of passive diffusion, active transport, and endocytosis. The paracellular pathway consists of the intercellular space and tight junctions, which are extremely impermeable to molecular and ionic flux and are the main barrier to paracellular transport. The barrier between urine and blood has been shown to have the highest recorded transepithelial resistance of all epithelia, with a value of up to 78,000 Ωcm^2^ (Lewis and Diamond 1976), and the urothelium of the urinary bladder is consistently the least permeable epithelium among most organisms (Lasič et al. 2015).

**Fig. 1:**
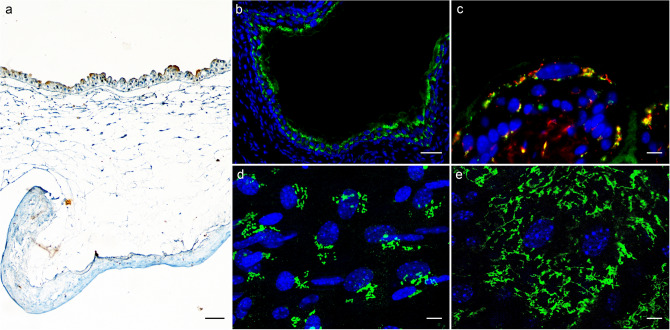
Normal porcine urothelium in vitro and mouse urothelium in vivo and the organization of the Golgi complex in urothelial cells. **a** Normal porcine UCs grown on a scaffold of human amniotic membrane and labeled with antibodies against uroplakins. The strongest labeling of differentiation-associated markers uroplakins (*brown*) is seen in the superficial UCs (umbrella cells). **b** Distribution of GRASP 55 (*green*) in mouse urothelium. **c** Co-localization of GRASP 55 (*green*) and giantin (*red*) in mouse urothelium. **d** Perinuclear distribution of GRASP 55 in intermediate UCs. **e** GRASP 55 distribution over the entire cytoplasm in the umbrella cell. Note: **a** Immunohistochemistry on formalin-fixed paraffin section. **b**, **c** Immunofluorescence on cryo-semithin sections (thickness 300 nm) prepared using the Tokuyasu technique. **d**, **e** Immunofluorescence on optical section of the UCs in the direction of the lumen—perpendicular to the apical surface.* Scale bars*, 50 µm (**a**, **b**); 10 µm (**c**, **d**, **e**)

The urothelium consists of a single layer of small basal urothelial cells (UCs), one to several layers of intermediate UCs, and a layer of highly differentiated superficial UCs, also known as umbrella cells (Fig. 1a). The latter are densely packed with cytoplasmic organelles, including Golgi apparatus and associated vesicles (Jost et al. 1989). Differentiation of superficial UCs follows the expression sequence of tight junction proteins (Varley et al. 2006; Smith et al. 2015; Kreft et al. 2005; Višnjar and Kreft 2013), surface glycans (Zupančič et al. 2014), apical membrane lipids, and the most abundant transmembrane proteins UPs in the apical plasma membrane of UCs (Fig. 1a) (Wu et al. 1994; Yu et al. 1994). UPs form urothelial plaques in the apical plasma membrane facing the lumen of the urinary bladder (12-nm-thick membrane), which are separated by hinges (5 to 7-nm-thick membrane) (Wu et al. 1994). Besides well-developed tight junctions, the UPs contribute to the permeability barrier through their structural organization (Lobban et al. 1998; Kachar et al. 1999; Liang et al. 2001; Min et al. 2003) (Liang et al. 2001; Kachar et al. 1999; Min et al. 2003; Lobban et al. 1998), including through the specific composition and position of a tyrosine-based motif in cytoplasmic domains of UPIa (YTML) and UPIIIa (YTSV) (Kreft et al. 2009a), and by hindering constitutive endocytosis from the apical plasma membrane (Kreft et al. 2009a; Tratnjek et al. 2017). Although UPs have been studied primarily in the mammalian urothelium, they may actually serve conserved and ancient functions in oocyte fertilization and only later acquired their ability to form two-dimensional crystals of 16-nm particles (urothelial plaques) during mammalian divergence, to perform additional functions, including the expansion of superficial UCs and the formation of a highly efficient permeability and mechanical barrier to protect and functionally modify the apical surface of the modern mammalian urothelium (Garcia-Espana et al. 2006; Desalle et al. 2014; Liao et al. 2018).

The Golgi complex is tightly integrated into the urothelial cellular system, where, like in other eukaryotic cells, it plays vital functions in the processing and sorting of proteins and lipids and serves as a signaling and a microtubule-organizing center. Here we will review the currently known contribution of the Golgi complex to the formation of the blood–urine barrier, mainly through its association with UP traffic in normal urothelial cells and its possible involvement in the loss of the blood–urine barrier in cancer cells. We will discuss a number of open questions about the structure and function of the Golgi complex in urothelial cell biology.

## Dynamic nature of the Golgi complex

As a central hub in the biosynthetic and endocytic pathways that define the urothelial permeability barrier, the Golgi complex constantly receives the flow of cargos and serves as a major processing station in the cell. Due to its dynamic nature, a sophisticated and constantly remodeling mechanism must be established to maintain the architecture and function of the Golgi complex for the uninterrupted transport of proteins and lipids.

In eukaryotic cells, the organization of the Golgi complex varies, although the proteins involved in the formation of the typical Golgi structure are similar. These proteins seem to differ only in the number of their isoforms. Therefore, the consensus is that different forms of the Golgi complex have formed from a single ancestor during evolution (Mowbrey and Dacks 2009), and thus all Golgi complex forms are the result of the different expression of the Golgi proteins (reviewed in (Mironov et al. 2017)).

There are three main morphological forms of the Golgi complex: (1) tubular networks (e.g., in microsporidia); (2) isolated perforated disks (e.g., in* Saccharomyces cerevisiae*); and (3) partially perforated disks organized in stacks (e.g., in other cells). The number of disks in a stack is in average less than eight (reviewed in Table 2 in the reference (Mironov et al. 2017)). Dozens, or even hundreds, of Golgi stacks in mammalian cells are interconnected into a ribbon-like structure that can act as a single organelle, with alternating compact (i.e., stacked cisternae) and non-compact (i.e., tubular–reticular) sections (Mironov and Beznoussenko 2011). Abundant evidence has been accumulated that a well-organized Golgi complex structure is required for its proper functions (Zhang 2021). However, the ribbon can undergo distinct disassembly processes that reflect the cellular state or extracellular/environmental signals and stress. The most dramatic Golgi complex change occurs in mitosis, when the ribbon is disassembled into vesicles and small non-polarized ministack (Mironov and Beznoussenko 2011) by a combination of unbundling of the ribbon, disk/cisternal unstacking, and vesiculation. In addition, the ribbon can also be fragmented to varying degrees during the progression of disease or cell death, or it may be detached/fragmented and positioned at specific cellular sites to provide additional functions during differentiation (Wei and Seemann 2017).

Such differentiation-dependent Golgi remodeling is observed also in UCs. The localization of Golgi-related markers [GM130 (*cis*-Golgi matrix protein of 130 kDa), GS15 (Golgi Snare 15 kDa), GS28, Golgin 97, GRASP55 and giantin], shows that in non-differentiated, UP-negative UCs the Golgi complex is mostly organized as a single ribbon-like structure close to the nucleus, whereas in differentiated, UP-positive UCs the Golgi complex is fragmented and spread almost through the entire cell (Fig. 1b–e). This phenomenon is observed in in vitro as well in vivo models. The role of such remodeling the Golgi complex in the biology of UCs and the formation of the blood–urine barrier will be discussed in the next sections.

## Golgi complex remodeling enables delivery of specific urothelial differentiation cargoes to the plasma membrane and the formation of the blood–urine barrier

### The involvement of the Golgi complex in the modification of UPs

Although the urothelium is a multifunctional epithelium also involved in sensory transduction that enables bladder fullness sensation and proper micturition (Khandelwal et al. 2009; Winder et al. 2014; Marshall et al. 2020; Dalghi et al. 2021), its maintenance of the blood–urine barrier is its most crucial function. That Golgi complex is the site of assembly of the thick luminal, i.e., apical plasma membrane of the superficial UCs, which makes a key contribution to the blood–urine barrier, was already suggested by Raymond Marian Hicks (Hicks 1966), who found similar thick patches of membranes (then termed asymmetric unit membrane, AUM) in the walls of the Golgi cisternae and in the apical plasma membrane. This implied the membrane flow from the Golgi complex to the cell surface. This and subsequent studies proposed that the membrane is transported to the luminal surface in the form of round- to discoid-shaped vesicles, derived from the Golgi cisternae, which fuse with, and become part of the apical plasma membrane (Severs and Hicks 1979; Alroy et al. 1982; Koss 1969; Hicks 1966). This hypothesis was later tested checked in several studies, which demonstrated that UPs are synthesized in the endoplasmic reticulum (ER) where they form two types of heterodimers (UPIa/UPII and UPIb/UPIIIa) before they exit the ER (Tu et al. 2002). UP-heterodimers are then transported from the ER to the Golgi complex (Višnjar et al. 2017), where Golgi-associated glycosylation reactions may exhibit UP-changes. Although GA-associated glycosylation reactions are clearly demonstrated in different cell types and animal species (Roth 1996), this process is poorly known for Golgi complex in UCs. It is identified that UPIb isolated from mouse and human urothelial plaques, and UPIIIa isolated from mouse, cattle, and human urothelial plaques contain complex glycans (Xie et al. 2006; Malagolini et al. 2000). The involvement of Golgi complex in the modification of UPs is also supported by the observation that the prosequence of UPII can be cleaved by the Golgi-protease furin (Hu et al. 2008). However, understanding the relationship between Golgi-associated glycosylation and Golgi structure is still enigmatic. Nevertheless, insights into the mechanisms of Golgi complex biogenesis from ribbon-like to fragmented form in UCs have been revealed in the last decade.

### The Golgi complex biogenesis from the ribbon-like to fragmented form

The functional reorganization of the Golgi complex is accompanied by specific rearrangements of the microtubules and intermediate filaments, which coincide with the differentiation of the UCs. The differentiation-dependent fragmentation of the Golgi complex and the subsequent spreading of Golgi to the cell periphery represent one of the key events that promote the uniform delivery of UPs throughout the apical plasma membrane of differentiating UCs and is thus of great importance for the final proper formation and maintenance of the blood–urine barrier (Kreft et al. 2010a). In FRAP experiments, the prominent recovery of fluorescently marked GalT into the bleached area was observed in UCs with ribbon-like Golgi, indicating that GalT could exchange between Golgi stacks within the ribbon-like Golgi. In UCs with transitional or fragmented Golgi, weak (i.e., approximately one order of magnitude slower in fragmented Golgi compared with ribbon-like Golgi) or no FRAP fluorescence into the Golgi region was found. Thus, in differentiated UCs, FRAP of GalT–GFP is inhibited, which suggests that Golgi fragments are functionally separated (Kreft et al. 2010a). Whether this applies to all Golgi proteins or only to GalT-GFP and whether the condition is the same in vivo, is currently not yet known.

Importantly, during such Golgi complex remodeling, it remains functional. A generation of specific UP cDNA constructs made it possible to study the dynamics of UPIb/UPIIIa transport in living UCs. Using cell transfection, time-lapse microscopy, immunohistochemistry and freeze-fracture replica immunolabeling, the biosynthesis and transport of UPs were confirmed in UCs and also in those cells that do not endogenously synthesize UPs and primarily have a ribbon-like Golgi complex (Višnjar et al. 2017). This study demonstrated the direct effect of UPs expression on Golgi complex fragmentation, allowing Golgi-outposts to be distributed as close as possible to the sites of cargo delivery at the plasma membrane (Višnjar et al. 2017). In post-Golgi compartments the 16-nm UP particles composed of six heterotetramers of UPIa/UPII-UPIb/UPIIIa (Kachar et al. 1999) gradually arrange into semi-crystalline urothelial plaques (Hudoklin et al. 2011, 2012; Warren and Hicks 1970; Severs and Hicks 1979). Freeze-fracture images revealed post-Golgi compartments, namely UP-positive discoidal or fusiform-shaped vesicles (DFVs) in close association with the Golgi and apical plasma membrane. Since the size of urothelial plaques in the membrane of DFVs resembles those found in close proximity to larger plaques in the apical plasma membrane, these associations are thought to be ideally configured to function in intracellular synthesis and transport as well as cytoplasmic-plasmalemmal transfer and also the continuous progressive incorporation of UP particles into urothelial plaques in the apical plasma membrane (Kreft and Robenek 2012).

### Transport of urothelial cargoes from the Golgi complex to the plasma membrane

Analysis of the Golgi-derived structures that transport UPs to the plasma membrane revealed that neither COPI nor COPII nor clathrin are present on their surface (Višnjar et al. 2017). Presumably, COPI-dependent vesicles are too small for the transport of large cargo aggregates including proteinaceous membrane thickenings in UCs (Mironov et al. 2017). This suggests that UP-containing structures likely utilize post-Golgi trafficking mechanisms used by a number of constitutively secreted cargo proteins (Polishchuk et al. 2003; Luini et al. 2008) and was indicated in the in vivo studies (Hudoklin et al. 2011, 2012). One of the common features of these mechanisms is the movement of post-Golgi carriers along microtubules and actin filaments. We have demonstrated the co-localization of microtubules and actin filaments with UPIb/UPIIIa-EGFP-positive vesicles and confirmed that UPIb/UPIIIa-EGFP transport depends on microtubules and actin filaments by determining their velocity and using depolymerization approaches. UP-trafficking mechanism currently proposed for still differentiating UCs is the following: UPIb/UPIIIa-EGFP-positive vesicles initially move from the Golgi complex to the plasma membrane along microtubules that enable transport at higher velocity. However, closer to the plasma membrane, UPIb/UPIIIa-EGFP-positive vesicles transition to actin filaments, which enable lower transport velocity and thus prime UPIb/UPIIIa-EGFP-positive vesicles for targeting to the plasma membrane (Višnjar et al. 2017).

When UCs become terminally differentiated with the bulk of UPs already at the apical plasma membrane, the microtubules, actin filaments and intermediate filaments are reorganized (Tratnjek et al. 2017; Kreft et al. 2009b, 2005, 2010a; Romih et al. 2002, 1999). Microtubular organization in the basal, central and subapical cytoplasm of highly differentiated UCs is diminished and actin filaments are mainly removed from the subapical cytoplasm (Tratnjek et al. 2017). This probably also contributes to the impeded endocytotic events. In contrast, trajectorial cytokeratin network accumulate in the apical cytoplasm (Kreft et al. 2002; Veranic and Jezernik 2002; Veranic et al. 2004), probably preventing the microtubular-dependent centralization of Golgi and maintaining the peripheral fragmentation of the Golgi complex in differentiated UCs. Currently, it is still not known, how the UP-trafficking machinery drives Golgi fragmentation in UCs and which transcriptional program and epigenetic changes are required for its reorganization.

In addition to UPs, the urothelium also has numerous surface receptors and ion channels that affect membrane conductance and are also considered in the context of maintaining the blood–urine barrier. The apical plasma membrane of the urothelium contains sodium, potassium, and calcium channels, as well as other cation channels. The basolateral plasma membrane of the urothelium also contains sodium and potassium channels as well as chloride channels, Na + /H + and Cl//HCO3 exchangers, and ATPase pumps (reviewed in (Lasič et al. 2015; Dalghi et al. 2020). Along with mechanosensitive ion channels, the urothelium contains many other receptors, e.g., the transient receptor potential (TRP) cation channels, which may play a role in the mechano- and/or chemosensory function of bladder urothelium (Winder et al. 2014). More specifically, TRPV4 plays a role in the transduction of intravesical mechanical pressure and thus bladder function (Gevaert et al. 2007). This channel as well as other TRP channels are gaining attention as possible new drug targets for the treatment of different urinary tract and bladder pathophysiologies (Lasič et al. 2015). Apart from the TRP channels, the urothelium receives many inputs through a vast number of other surface receptors and ion channels such as nicotinic and muscarinic receptors, the purinergic P2X family of ATP receptors, TRAAK and TREK-1 channels, acid-sensitive ion channels (Wang et al. 2005; Khandelwal et al. 2009; Araki et al. 2008; Birder and Andersson 2013), and the mechanosensitive ion channel PIEZO2, which it is required for low-threshold bladder-stretch sensing and urethral micturition reflexes (Marshall et al. 2020). However, the transport of these surface receptors and ion channels through the Golgi complex to the plasma membrane of UCs and their exact ultrastructural localization in the plaque or hinge regions of UCs is not known. The possible role of the Golgi complex in their formation and perhaps even their reciprocal effect on the Golgi complex itself have also not been explored. The relationship between the Golgi complex and the tight junctional proteins has also not yet been investigated.

## Fragmentation of the Golgi complex enables long distance urothelial cell–cell communication

Communication between cells is crucial for unicellular organisms, especially when they are forming a multicellular structure, like a biofilm, and for all multicellular organisms (Dubey and Ben-Yehuda 2011). Tunneling membrane nanotubes (TNTs) are membrane protrusions connecting nearby or distant cells in vitro and in vivo. Since their discovery in 2004, TNTs have gained much attention as critical players in intercellular communication (Rustom et al. 2004). They mediate intercellular exchange of cargoes including proteins, RNAs, miRNAs, ions, bacteria, viruses, prions, and organelles between connected cells (Vignais et al. 2017).

TNTs transport organelles such as lysosomes, mitochondria, the endoplasmic reticulum and Golgi complex (Antanavičiūtė et al. 2014; Zhang et al. 2021; Wang et al. 2011). Recently, it has been shown that the location of the centrosome not only predicts the site of TNT formation but can also facilitate intercellular transport by regulating microtubule formation and Golgi orientation (Dubois et al. 2021). We have found that Golgi outposts are also seen in normal and cancerous UCs (Resnik et al., in submission), suggesting Golgi fragmentation in the context of urothelial cell–cell communication.

Since TNTs are very sensitive to mechanical stress and chemical fixation, many basic properties of TNTs are still poorly known. Correlative phase contrast and fluorescence microscopy have shown that less than 30% of TNTs present in living cells are retained after the immunolabeling procedure (our unpublished data). Nevertheless, the mechanism and nature of Golgi fragmentation in TNT remain to be explored.

## Contribution of the Golgi complex to the loss of the blood–urine barrier in cancer

Bladder cancer is seventh most common cancer in men. Its epidemiology still shows a clear male predominance, and incidence and mortality rates differ per European country, probably due to differences in risk factors, detection, and availability of treatments (Witjes et al. 2021). On the global level, incidence and mortality rates vary due to different methodologies and diagnostic practices. Approximately 25% of patients with bladder cancer present with muscle-invasive or metastatic disease, others have non-muscle invasive bladder cancer (NMIBC) (Taskovska et al. 2020).

Even the first, epoch-making studies of the bladder epithelium using light and electron microscopy and histochemistry showed that the bladder is excellently adapted to urine and its contents. On the other hand, the urothelium bears the brunt of the attack by pollutants and various oncogenes. In 1978, Melicow assumed that the ongoing battle may be of long duration or recurrent and influence on specialized UC apical plasma membrane and the Golgi complex (Melicow 1978). In this chapter, we discuss the possible indirect role of the Golgi complex in the increased transcellular permeability of the cancerous urothelium. We will not refer here to the loss of the blood–urine barrier due to the loss of the tight junctions and the increased paracellular permeability, which is one of the first steps that could lead to the invasion of cancer cells into the blood vessels (Jerman et al. 2021).

It is known that the luminal membrane is changed in cancer UCs and, instead of urothelial plaques, a thinner, smooth, flexible membrane develops with a filamentous glycocalyx above it (Melicow 1978; Resnik et al. 2015; Yu et al. 2016; Zupančič et al. 2014, 2011; Lobban et al. 1998; Olsburgh et al. 2003). Remarkably, altered glycosylation has been a hallmark of most cancer cells (Zhang 2021). To understand the causes of altered Golgi structure and function in bladder cancer, efforts must be made to characterize the structural proteins of the Golgi under physiological and pathological conditions.

When we transfected HeLa cells with UPIb-EGFP or UPIb/UPIIIa, which do not endogenously express UPs, the Golgi complex was fragmented (Višnjar et al. 2017). During urothelial carcinogenesis, UCs lose expression of UPs (Lobban et al. 1998; Zupancic and Romih 2013) and fragmentation of the Golgi complex in muscle-invasive bladder cancer cells T24 is not achieved (our unpublished data). This differs from descriptions in other cell types and disease states. It is known that a fragmented Golgi ribbon is commonly associated with many stress and pathological conditions, including apoptosis (Machamer 2015; Lane et al. 2002), bacterial or viral infections (Jimenez et al. 2016; Lavi et al. 1996), Alzheimer’s disease (Joshi et al. 2014, 2015), Parkinson’s disease (Rendón et al. 2013; Tomás et al. 2021) and various forms of cancer (Núñez-Olvera et al. 2020; Petrosyan 2015). Despite similar phenotypic characteristics among these diseases, the mechanisms that cause Golgi fragmentation and dysfunction can range from imbalanced membrane flux, altered microtubule dynamics, to post-translational modifications or irreversible proteolytic cleavage of Golgi structural proteins. It is not clear whether the mechanisms that drive Golgi ribbon disassembly in mitosis or during differentiation are also underpinning Golgi fragmentation during disease progression. In fact, the correlation between the observed morphological alterations and dysfunction of the Golgi complex is often unclear, as the fragmentation may directly cause, partially contribute to, or merely be the outcome of pathology (Wei and Seemann 2017).

Golgi phosphoprotein 3 (GOLPH3) is a highly conserved peripheral membrane protein of ~ 34 kDa that is localized in the* trans*-Golgi network and dynamically exchanges with a large cytosolic pool (Wu et al. 2000; Kuna and Field 2019; Cavieres et al. 2020). GOLPH3 plays a role in anterograde and retrograde Golgi trafficking and is considered as a hub for regulating the Golgi complex organization and function. It is also considered as the first oncoprotein of the Golgi complex to play an important role in various types of cancer (Yu et al. 2020), including bladder cancer (Zhang et al. 2015). Overexpression of GOLPH3 had significant correlation with poorer survival for bladder cancer patients. The action of GOLPH3 probably occurs via several mechanisms, including activation of PI3K/AKT/mTOR pathway (Zhang et al. 2015). Its inhibition in cells overexpressing GOLPH3 could therefore be a therapeutic approach in cancer.

The association of GOLPH3 with Golgi membranes depends on its binding to phosphatidylinositol-4-phosphate (PtdIns4P), which promotes the exit of vesicles in the Golgi for transport to the plasma membrane. PtdIns4P is enriched in the* trans*-Golgi and therefore recruits GOLPH3. This allows the formation of a GOLPH3 complex, which is formed when GOLPH3 binds to myosin18A, which in turn binds F-actin. This complex generates a pulling force to extract vesicles from the Golgi; interference with this GOLPH3 complex leads to a drastic reduction in vesicle trafficking (Kuna and Field 2019). Since GOLPH3 plays a role in anterograde and retrograde Golgi trafficking and is involved in vesicle budding from the* trans*-Golgi and in several post-Golgi protein trafficking events, it may also be involved in UP trafficking to the plasma membrane of UCs. However, this mechanism has not yet been explored in UCs. In conclusion, knowledge of the molecular mechanisms involved in alterations to the urothelial Golgi complex phenotype under pathological conditions is limited but urgently needed to decipher the pathogenesis of various urinary tract diseases and to develop new therapeutic approaches.

## Summary and open questions

**Fig. 2:**
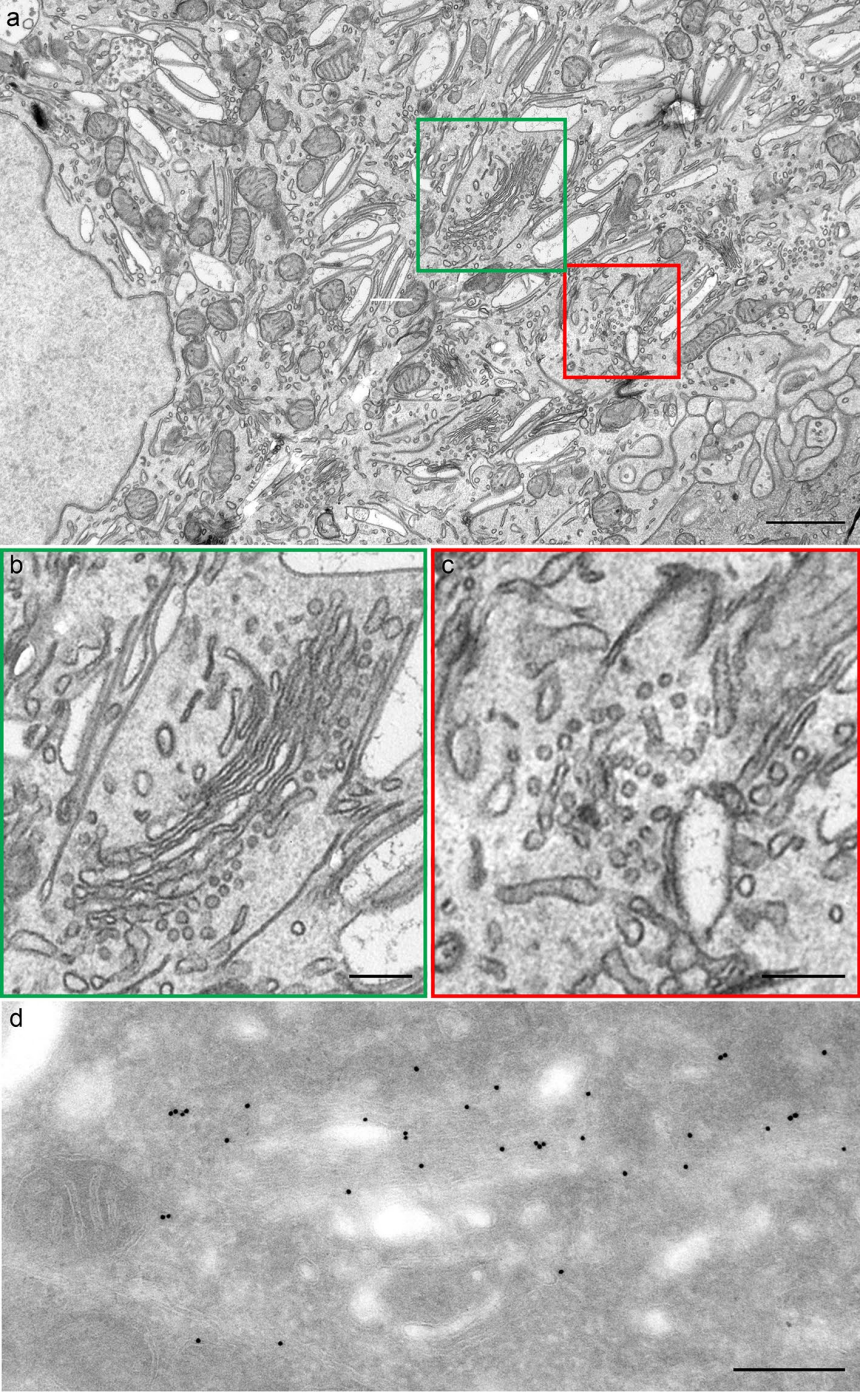
Representative examples of the Golgi complexes from umbrella cells. **a** Low magnification of the umbrella cell. The* green box* indicates the Golgi complex. The* red box* shows the ER exit site. **b** Enlargement of the area inside green box in (**a**). **c** Enlargement of the area inside the red box in (**a**). **d** Immuno EM based on cryosections according to Tokuyasu. Labeling for GRASP55 is present over Golgi cisternae.* Scale bars* 600 nm (**a**); 220 nm (**b**); 180 nm (**c**); 90 nm (**d**)

Many of the pathways to the apical and basolateral UC surfaces involve transient passage through the Golgi complex. While recent studies significantly advanced our understanding of how the Golgi complex is involved in UP-transport to the plasma membrane and as such is critical for the formation and maintenance of the blood–urine barrier, it is still not known how the UP-transport machinery drives Golgi-fragmentation in UCs and what transcriptional programs and epigenetic changes are required for the Golgi complex reorganization. Moreover, it looks like there are different types of Golgi fragmentation in UCs, depending on what cell cycle phase the cell is in and what stage of differentiation it is in. If we look at the morphology of the Golgi complex in highly differentiated superficial UCs (umbrella cells) (Fig. 1e and Fig. 2a, b, d), the Golgi complex is still very active, so its fragmentation is not the same as in mitosis.

**Fig. 3:**
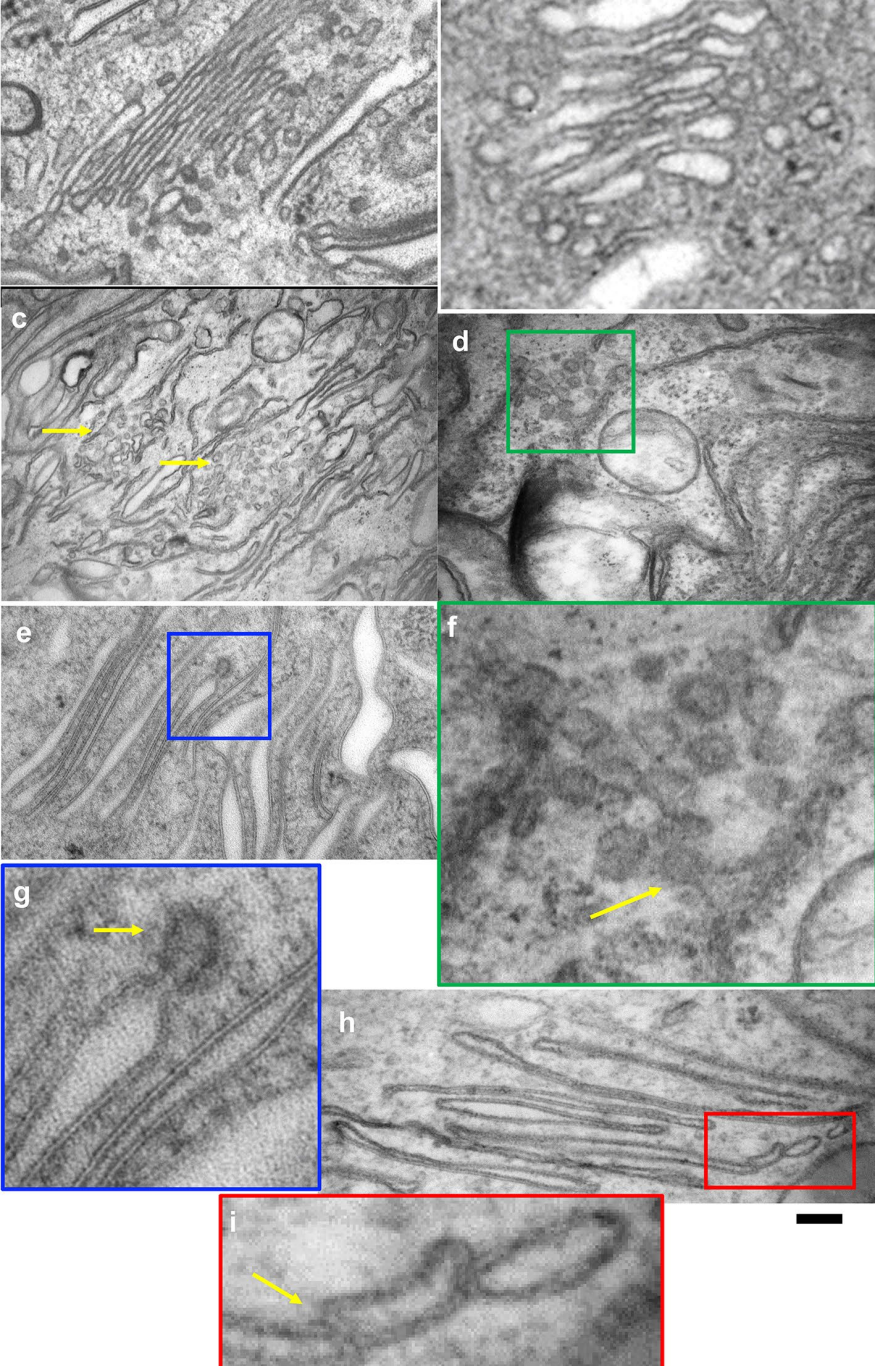
Structure of the Golgi complex and its derivatives in umbrella cells. **a** The Golgi complex with the presumably COPI-dependent vesicles only at the Golgi* cis*–side. **b** Ministack with COPI vesicles at all levels. **c**, **d** ER exit sites (*yellow arrows* in c;* green box* in d). **e** Clathrin-coated bud on the FV. **f** Enlargement of the area inside green box in (**d**). The* yellow arrow* shows COPII-coated bud. Deformation of round profiles typical for other cell types. **g** Enlargement of the area inside the blue box in (**e**). **h** Image shows tight attachment of post-Golgi transport vesicle to the FV. **i** Enlargement of the area inside the red box in (**h**). The* yellow arrow* shows attachment of post-Golgi transport vesicle to the FV.* Scale bars* 220 nm (**a**, **c**, **d**); 110 nm (**b**); 200 nm (**e**, **h**)

There are several additional unclear problems. It is not clear how the UPs are transported from the ER to the Golgi complex. In umbrella cells, there are ER exit sites (ERES) (Fig. 2c and Fig. 3c, d, f). However, the sections of these compartments are not round and the round profiles which usually are observed within ERES are not seen. Part of their limiting membrane appears rather plane. These membranes are similar to membranes of the FVs, although these membranes were not coated with glycocalyx similar to that observed on the luminal surface of the plaque membranes of FVs and the apical plasma membrane (Fig. 3e, g, h). This may be the result of not yet formed and/or non-aggregated UP particles along with non-glycosylation. The urothelial plaques are covered by glycocalyx. There is less glycocalyx in the urothelium of mice than in the urothelium of humans and pigs (please see Fig. 3 in the reference (Kreft et al. 2010b) and Fig. 6 in the reference (Jerman et al. 2014)). This difference could be due to physiological and structural differences between the species. It could be that the glycocalyx is intensively formed as the first line of defense against urine permeability, which is later achieved and maintained in the animals with more concentrated urine by a high density of UP particles in superficial urothelial cells. The UP particles contribute to the apical plasma membrane region (also called urothelial plaque or asymmetric unit membrane (AUM)) being thicker than in the hinge region (see Fig. 2B in the reference (Hudoklin et al. 2012)). It is believed that the thicker E-half of the apical plasma membrane is mainly due to the specific structure of UPs with the bulk of extracellular part. Nevertheless, the presence of ERES in the umbrella cells suggests that there occurs intensive synthesis of membranes and that these membranes are not necessary for mitotic division but for the generation of FVs.

In umbrella cells, the Golgi complex contains COPI-vesicles near its rims (Fig. 3a, b). The urothelial plaques consist of two-dimensional (2D) crystals of hexagonally packed 16 nm UP particles (Min et al. 2003). Therefore, aggregates of two such particles are already incompatible in size with COPI-dependent vesicles. Moreover, near-Golgi vesicles are roundish (Fig. 3h, i) in contrast to the round profiles near ERES (Fig. 3d, f). This suggests that COPI vesicles are not involved in the transport of UP particles through the Golgi complex. On the other hand, it is known that COPI vesicles do not participate in the recycling of Golgi resident proteins (Mironov and Beznoussenko 2019; Beznoussenko et al. 2022) and UP particles are rather big for diffusion along the membrane continuum within Golgi cisternae.

During maturation of umbrella cells, the Golgi complex becomes larger. The GM130-positive compartment unites the Golgi stacks and the* trans*-most cisterna (see Fig. 2 of (Hudoklin et al. 2009)). This situation is similar to the situation in neurons, where the* cis*-most cisterna joins the Golgi stacks and is the opposite to the situation in fibroblasts, where the * trans*-most cisterna joins the Golgi stack (Mironov et al. 2017; Beznoussenko et al. 2022). However, it seems that the Golgi complex contains only limited amount of UPs. This suggests that in umbrella cells, there is a stimulation of synthesis of Golgi enzymes and proteins of Golgi matrix.

When UPs were synthesized in MDCK cells, they formed spots which move peripherally. This process is mostly microtubule dependent (Višnjar et al. 2017). Thus, the appearance of UPs induces the interaction of post-Golgi vesicles and microtubules. The mechanisms of this process are unknown. We have found that more glycocalyx is present in these vesicles when UCs are not terminally differentiated, which is usually the case when UCs are grown in vitro (see Fig. 1C, F, K in the reference (Predojević et al. 2022).

Uroplakins are glycosylated and pass through the Golgi complex (see Fig. 3 by (Višnjar et al. 2017). However, further ultrastructural studies are needed to analyze these proteins within the Golgi cisternae. It could also be that UP-positive membranes are present near the Golgi complex being connected with it. Thus, a further 3D analysis of the Golgi complex is highly necessary.

In conclusion, the Golgi complex plays an important role in the UC differentiation, however there is still question what factors are responsible for the differentiation associated interplay between the Golgi complex and UCs. Nevertheless, there is a growing awareness that the Golgi complex plays an important role in precisely tuned blood–urine barrier machinery within the bladder urothelium and may also play a role in a variety of bladder diseases. This therefore needs to be considered in future research and in the development of more effective disease treatment.

## Data Availability

Authors can confirm that all relevant data are included in the article.
